# Color of Lightning

**Published:** 1881-08

**Authors:** 


### COLOR OF LIGHTNING.

The color of lightning is altogether due to the nature of the substance which is made incandescent in its track. The blue, red, purple or silver tints, which are ordinarily much more brilliantly marked in warm climates and intertropical countries than they ever are in England, are due to the same circumstances as the color which is designedly communicated to the light of different kinds of fireworks.

It is a result of the intrinsic natures of the vaporized particles which are made to shine. The vapor from iron has one kind of sheen and the vapor of sulphur another. Each different foreign ingredient that floats in the air has its own proper hue, which it can communicate to the lightning.

The broad flashes of light that appear in the clouds during a thunder-storm, and that are distinguished as sheet-lightning, are very often merely the reflections from the cloud mist of the discharges from one part to another with each redistribution of the internal charge, as the tension at the outer surface is changed by an external flash.

This redistribution of the internal charge is sometimes also marked by very beautiful lines of corruscation playing upon the dark background as the storm drifts away.

There is a table mountain a few miles away from Pietermaritzburg, in Natal, over which this kind of display is continually exhibited.

The retreating storm-clouds linger over the flat top of this mountain, where they can be seen from the city in the advancing night.

In this dark canopy of the mountain bright corruscations accompanying each redistribution of the electric charge can be watched for hours at a time—now assuming the form of coronals of electric fire, now running along in machiolated horizontal lines just above the flat top of the mountain, and now radiating out in all directions from a central loop, like the cracks of starred glass.

